# Anti-γ-aminobutyric acid-A receptor encephalitis with refractory seizures and cognitive impairment in a young woman: A case report

**DOI:** 10.3389/fneur.2022.954494

**Published:** 2022-08-29

**Authors:** Xue Yang, Bo Deng, Shengjun Wang, Xiaotang Wang, Lili Cao, Xiangjun Chen, Xiuhe Zhao

**Affiliations:** ^1^Department of Neurology, Qilu Hospital, Shandong University, Jinan, China; ^2^Department of Neurology, Huashan Hospital, Fudan University, Shanghai, China

**Keywords:** anti-GABA_A_R encephalitis, seizures, case report, encephalitis, neurology

## Abstract

Anti-γ-aminobutyric acid-A receptor (GABA_A_R) encephalitis is an underappreciated cause of autoimmune encephalitis and remains refractory to antiepileptic therapies unless autoimmune responses are addressed. Herein, we reported a case of anti-GABA_A_R encephalitis in a young woman. A 29-year-old woman was admitted because of seizures for 10 months, memory decline for 7 months, and paroxysmal limbs jerking for 5 months. At admission, the patient showed mild cognitive impairment. Cell-based assays found no antibodies associated with common autoimmune encephalitis in the cerebrospinal fluid (CSF) and no antibodies in the plasma and CSF against central nervous system demyelination-associated proteins. MRI revealed multiple cortical-subcortical abnormalities and electroencephalography demonstrated periodic epileptiform discharges during paroxysmal clonus. A second test 1 month after admission detected antibodies against GABA_A_R α1/β3/γ2 in the plasma and CSF, leading to a diagnosis of anti-GABA_A_R encephalitis. The patient received intravenous immunoglobulin, prednisone, azathioprine, and levetiracetam and recovered from limb jerks and was no longer amnesic. A second episode occurred after an apparent cold and was managed by intravenous immunoglobulin, cyclophosphamide, and methylprednisolone with subsequent prednisone and levetiracetam. The patient was able to speak and ambulate after 15 days of treatment. Her MMSE, MoCA, and MRS scores improved. Physicians should harbor a high index of suspicion of anti-GABA_A_R encephalitis in refractory encephalitis patients with the manifestation of seizures or psychiatric disorders. Tests for a comprehensive panel of antibodies associated with anti-GABA_A_R encephalitis should be carried out in suspected cases and immunotherapy should be promptly initiated upon diagnosis to prevent irreversible neurological damage.

## Introduction

Encephalitis with the manifestation of seizures or psychiatric disorders can result from autoimmune responses induced by antibodies against excitatory or inhibitory synaptic receptors or associated cell-surface proteins (1). The γ-aminobutyric acid-A receptor (GABA_A_R) is a ligand-gated chloride channel that mediates fast inhibitory synaptic transmission in the central nervous system (CNS) (2, 3). Antibodies to GABA_A_R have been associated with lengthy and refractory seizures (4). Seizures may be refractory to antiepileptic therapies unless the autoimmune responses are addressed, and epilepsy or recurrent seizures may impact cognitive ability (5). Therefore, it is critical that anti-GABA_A_R encephalitis be promptly recognized and treated in order to facilitate the recovery of neurological function. Herein, we reported a case of anti-GABA_A_R encephalitis in a young woman with refractory seizures, multifocal cerebral abnormalities, and positive GABA_A_R antibodies.

## Case presentation

A 29-year-old woman was admitted to the Neurology Emergency Department of our hospital on 8 July 2020 because of seizures for 10 months, memory decline for 7 months, and paroxysmal limb jerk for 5 months. The patient had two episodes of generalized tonic-clonic seizures with concurrent fever and headache in September 2019. Cerebrospinal fluid (CSF) examination revealed leukocytosis (16/mm^3^, reference range, 0–5/mm^3^), with 91% lymphocytes, 4% neutrophils and 2% eosinophils. In December 2019, she showed slowed response, impaired memory, and bradyphrasia (slowed speech). Two months later, paroxysmal myoclonic-like jerks appeared, successively involving the head, the left, and right arms. From September 2019 to February 2020, the patient was diagnosed with suspected autoimmune encephalitis at a local hospital and was treated with 3 cycles of intravenous immunoglobulin (400 mg/kg/d for 5 days) and 2 cycles of methylprednisolone (1,000 mg/d for 5 days). Antibodies associated with autoimmune encephalitis in the CSF were negative by cell-based assays on two occasions.

During physical examination at admission, the patient complained about recent insomnia and visual hallucinations. No remarkable physical findings were noticed. She scored 25/30 on the Mini-Mental State Examination (MMSE), 23/30 on the Montreal Cognitive Assessment (MoCA), and 2 on the Modified Rankin Scale (MRS), showing that the patient had mild cognitive impairment. A laboratory study showed elevated plasma ammonia at 41 mmol/L (reference range, 9–33 mmol/L), and the patient was positive for anti-rubella virus/cytomegalovirus/herpes simplex virus IgG. CSF cytology and biochemistry were within normal limits.

Cell-based assays were performed for antibodies against specific neuronal surface targets including NMDAR, AMPAR 1/2, LGI1, CASPR2, GABA_B_R, GABA_A_Rα1/β3, DPPX, GlyR α1, mGluR5, D2R, IgLON5, and neurexin-3α, but yielded no positive findings. No antibodies were detected in the plasma and CSF against central nervous system demyelination-associated proteins including AQP4, MOG, and GFAP. A complete mitochondrial genome high-throughput sequencing of whole blood cells revealed no pathogenic or suspected pathogenic mutations.

**Figure 1:**
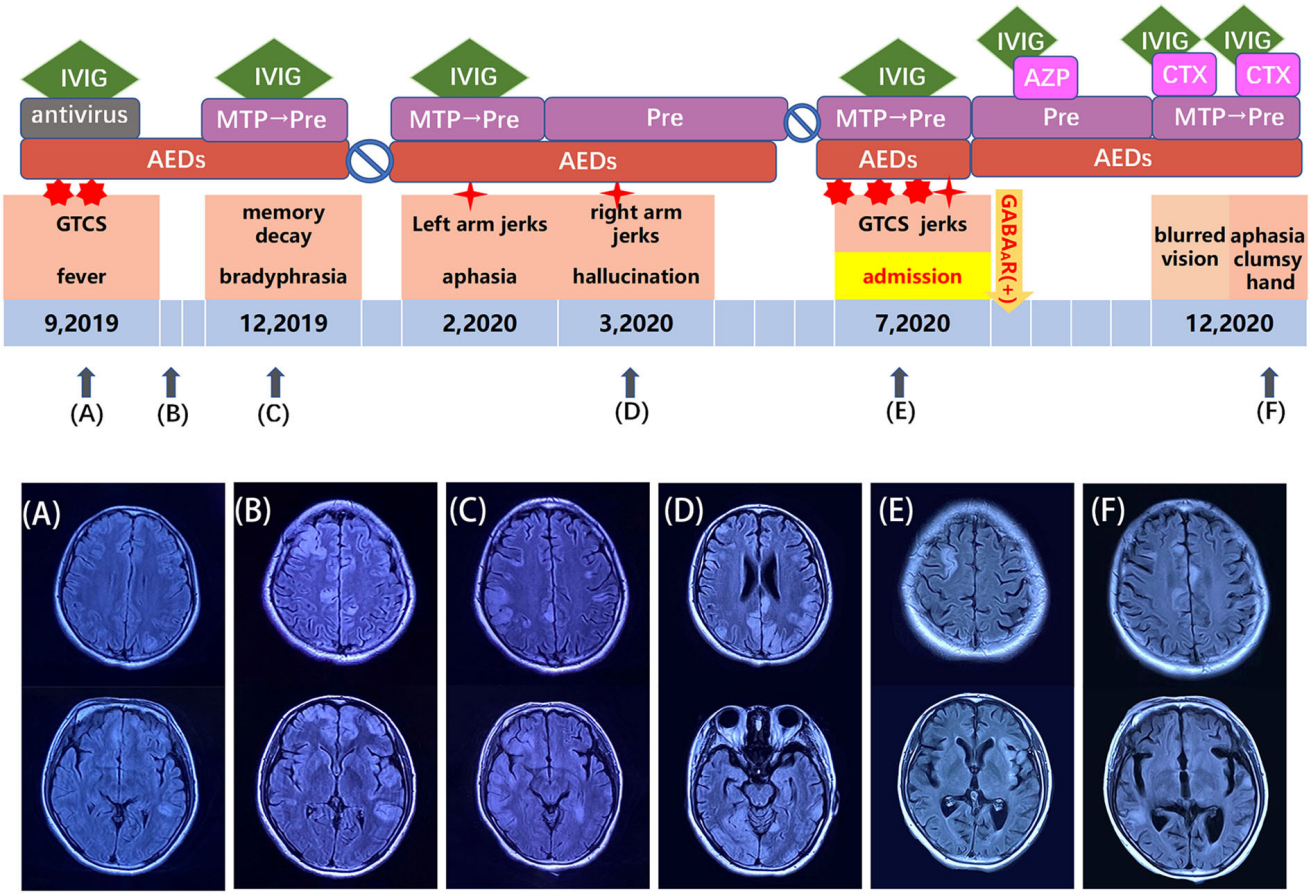
Schema for the disease course, treatments, and MRI manifestations of anti-GABA_A_R encephalitis in a 29-year-old woman. **(A–F)** Clinical manifestations and treatment options corresponding to each time point in the course of the disease. AEDs, anti-epilepsy drugs; AZP, azathioprine; CTX, cyclophosphamide; IVIG, intravenous immunoglobin; MTP, methylprednisolone.

**Figure 2:**
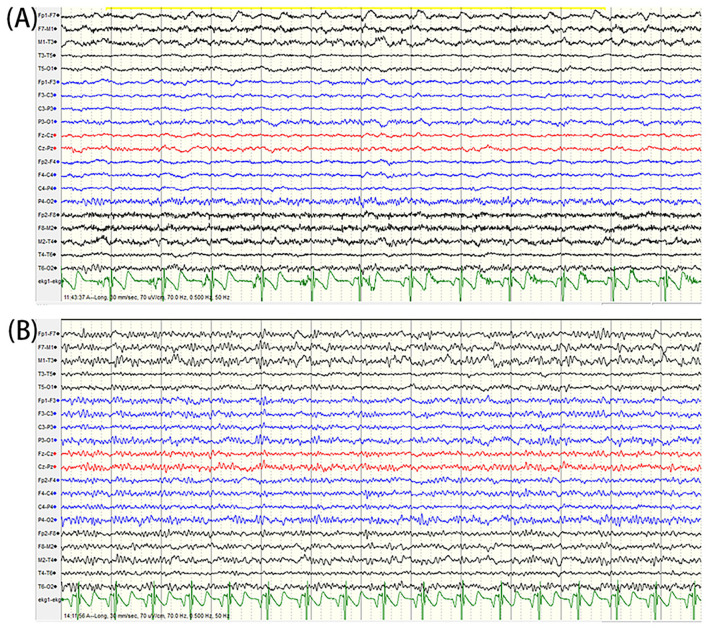
EEG manifestations at the time of right arm paroxysmal clonus occurrence **(A)** and after intravenous midazolam **(B)**.

**Figure 3:**
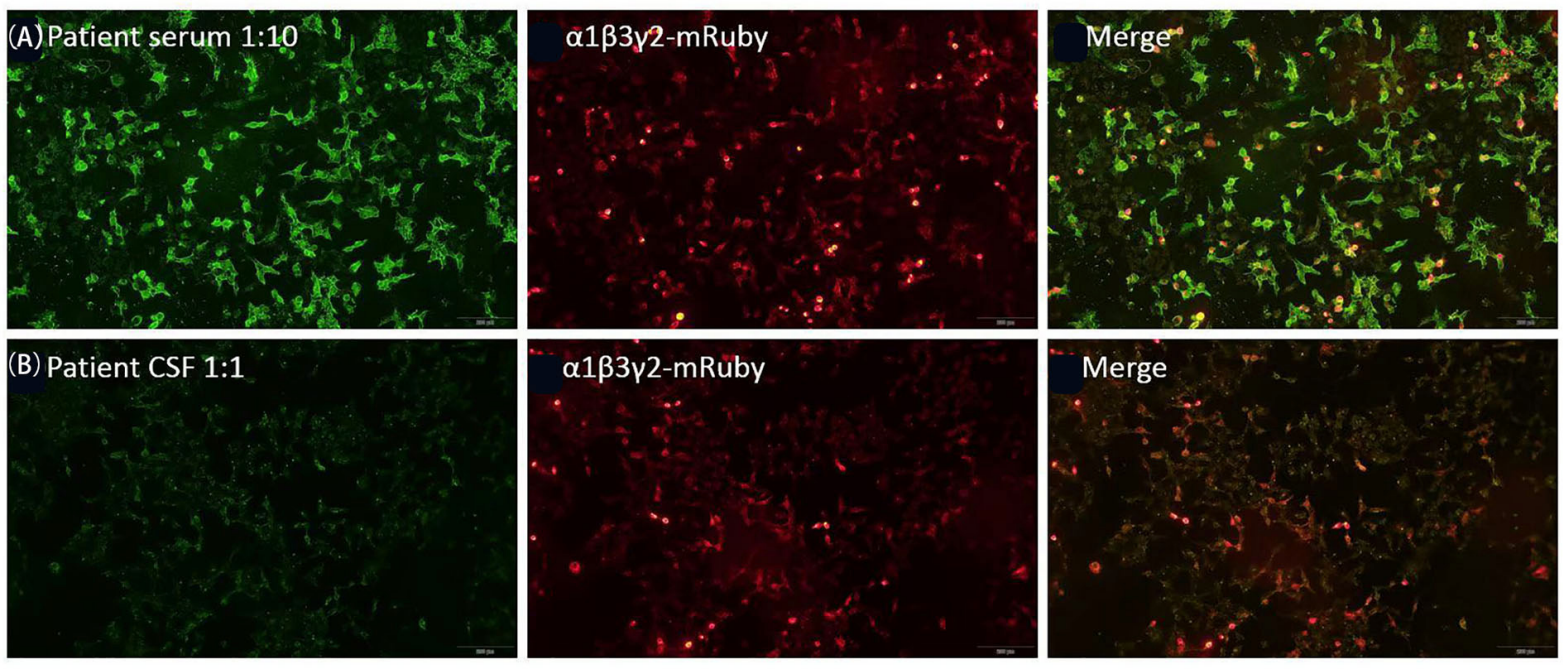
Reactivity of the patient's serum **(A)** and cerebrospinal fluid (CSF) **(B)** with live HEK293 cells expressing human α1/β3/γ2 subunits of GABA_A_R.

MRI of the brain using fluid-attenuated inversion recovery (FLAIR) revealed multiple, asynchronous, cortical-subcortical abnormalities in the frontal, temporal, parietal, occipital, and insular lobes, and mismatched cerebrovascular distribution (Figure 1). Electroencephalography (EEG) demonstrated periodic epileptiform discharges in chains lasting for 2 min in the left frontal region when right arm paroxysmal clonus occurred (Figure 2A), which was nearly synchronously attenuated by intravenous midazolam (Figure 2B) A second test was performed on August 12, 2020, for antibodies in the plasma and CSF against GABA_A_R α1/β3 (4) and γ2 subunits (6) using live HEK293 cells expressing α1/β3/γ2 subunit and was positive (Figure 3). The patient was diagnosed with anti-GABA_A_R encephalitis.

### Treatment

The patient was started with intravenous immunoglobulin (400 mg/kg/d for 5 days), prednisone (1 mg/kg/d PO), azathioprine (50 mg PO bid), and levetiracetam (0.5 g PO bid). One month later, azathioprine was withdrawn due to liver toxicities. At the outpatient follow-up visit in October 2020, the patient recovered from limb jerks and was no longer amnesic. In December 2020, following an occasional cold, she developed aphasia, with a clumsy right hand and blurred vision. Multiple cortical-subcortical T2/FLAIR MRI abnormalities appeared in the bilateral frontal and temporal lobes. Plasma and CSF GABA_A_R antibodies were positive. The patient was admitted to the hospital and treated with 2 cycles of intravenous immunoglobulin (400 mg/kg/d for 5 days), two cycles of cyclophosphamide (0.6 g twice a month), and 1 cycle of methylprednisolone (500 mg/d for 5 days) with subsequent oral prednisone (1 mg/kg/d). In addition, levetiracetam (0.5 g twice a day) was given. The patient could speak and ambulate after 15-day treatment. Her MMSE score was 27/30, her MoCA score was 27/30 and her MRS score was 1.

## Discussion

Gamma-aminobutyric acid is one of the most important inhibitory neurotransmitters and plays biological roles through ionotropic GABA_A_ receptors and metabotropic GABA_B_ receptors. The GABA_A_ receptor, which mediates fast-inhibitory neurotransmission in the brain as a pentamer in the order γ-β-α-β-α, has recently been identified as an autoantigen associated with limbic encephalitis (7). Antibodies to the α1 and β3 subunits of GABA_A_R with high serum and CSF titers were first reported in 6 patients with encephalitis and refractory seizures in 2014 (4) and later the β3 subunit was revealed to be the main target of plasma antibodies (8). Subsequently, the γ2 subunit was also found as a target for antibodies in autoimmune encephalitis (6). In the current case, antibodies to GABA_A_R were not detected on two occasions at the early stage by a specific GABA_A_R cell-based assay using live HEK cells expressing α1/β3 subunits. Plasma and CSF reactivities were demonstrated by HEK cells expressing α1/β3/γ2 subunits ~1 year later, allowing a final diagnosis of anti-GABA_A_R encephalitis. Therefore, we speculated that the omission of the γ2 subunit of GABA_A_R in the earlier assays may have led to missed diagnosis, suggesting that more attention should be paid to novel antibody subunit screening to avoid diagnostic delay in autoimmune diseases.

Anti-GABA_A_R encephalitis, which affects a very broad age range and both sexes, is characterized by severe seizures, cognitive impairment, consciousness decline, altered behavior, and movement disorders. Significantly, about 88% of the patients usually have seizures at presentation, which frequently progress to status epilepticus (9). In addition, lengthy and refractory epilepsia partialis continua are common. Children are more likely to develop generalized seizures than adults who predominantly develop focal seizures (4, 9, 10). Our case had an acute onset and suffered from generalized tonic-clonic seizures, partial seizures, and cognitive disorder, which were aggravated after drug discontinuation or rapid reduction. This is consistent with previous studies (4, 9), indicating the possibility of autoimmune disease.

Given the extensive and age-related disease spectrum of anti-GABA_A_R encephalitis, we speculate that there might be pathophysiological links between subunit specificity and symptoms. For instance, receptor internalization occurs for α1-specific GABA_A_R antibodies (8). Direct receptor activation or complement deposition may be induced in other subunit-associated encephalitides. It appears that emotional or behavioral disturbances tend to be the main clinical manifestations in patients with α1-specific antibodies, and learning disabilities or spatial disorientation with γ2-specific ones, except for seizures (6). In the current case, the patient suffered from frequent episodes of seizures and memory decline.

Previous studies suggested that 40% of patients with anti-GABA_A_R encephalitis have tumors, mostly thymomas, and less commonly, other neoplasms that may impair the immune system (9). Interestingly, coexisting antibodies (LGI1 or CASPR2) were detected in patients suffering from both anti-GABA_A_R encephalitis and thymomas (8, 9). Type 1 diabetes mellitus and/or Hashimoto's thyroiditis were also reported in some adult patients (4).

Multifocal unilateral or bilateral cortical-subcortical T2/FLAIR MRI abnormalities occur in 80% of patients, predominantly involving temporal and frontal lobes, but also basal ganglia, insular cortex, and other regions (8, 9, 11), which could asynchronously manifest during the disease (9). Interestingly, brain lesions tend to partly or completely vanish over weeks, leaving little or no residual findings after immune treatment (12).

The GABA_A_R antibodies cause a broad spectrum of symptoms, which seem less responsive to immunomodulatory treatment compared with other autoimmune encephalitides, and might be potentially lethal (6, 9). Therefore, prompt recognition and treatment of anti-GABA_A_R encephalitis are crucial to improving neurologic recovery in patients.

Moreover, anti-GABA_A_R encephalitis is characterized by multifocal and extensive brain MRI abnormalities. Our case showed that the immune response might have primarily contributed to cerebral damage. The distribution and severity of MRI abnormalities were inconsistent with the frequency and severity of seizures. In other autoimmune encephalitides, the MRI findings are often normal (NMDAR) (13), or predominantly involve the hippocampus (AMPAR, GABA_B_R, LGI1) (14, 15), in which the patients also suffer from lengthy and frequent seizures.

Our case met the basic clinical, imaging, and laboratory performance of anti-GABA_A_R encephalitis, and achieved a satisfactory effect to immunomodulatory treatment. Notably, omission of the γ2 subunit of GABA_A_R resulted in a diagnostic delay, suggesting that comprehensive detection of antibody subunits should be performed at the early stage of the disease. The transient mild elevation of plasma ammonia was observed with no abnormal findings of abdominal-pelvic CT scan in the course, which was possibly attributed to diet or medication. Otherwise, there were several differential diagnoses to consider, such as mitochondrial encephalopathy lactic acidosis and stroke-like episodes, anti-MOG associated encephalitis with seizures, and so on.

## Conclusion

In our case, although the patient was treated with several cycles of immunotherapy, recurrent neurological deficits occurred, and an MRI scan in December 2020 showed mild brain atrophy (Figure 1). We speculated that it might be related to rapid drug withdrawal and delayed immunosuppressive therapy before diagnosis. Therefore, suspected patients should be examined for a comprehensive panel of antibodies associated with anti-GABA_A_R encephalitis and immunotherapy should be promptly initiated upon diagnosis to prevent irreversible neurological damage.

## Data availability statement

The original contributions presented in the study are included in the article/Supplementary material, further inquiries can be directed to the corresponding author.

## Ethics statement

The studies involving human participants were reviewed and approved by all procedures involving the human participant were in accordance with the ethical standards of Ethics Committee in Qilu Hospital of Shandong University. The patients/participants provided their written informed consent to participate in this study. Written informed consent was obtained from the individual(s) for the publication of any potentially identifiable images or data included in this article.

## Author contributions

XY and XZ contributed to the study conception and design. All authors collected the data, performed the data analysis, contributed to the interpretation of the data, completion of figures and tables, drafting of the article, and final approval of the submitted version.

## Conflict of interest

The authors declare that the research was conducted in the absence of any commercial or financial relationships that could be construed as a potential conflict of interest.

## Publisher's note

All claims expressed in this article are solely those of the authors and do not necessarily represent those of their affiliated organizations, or those of the publisher, the editors and the reviewers. Any product that may be evaluated in this article, or claim that may be made by its manufacturer, is not guaranteed or endorsed by the publisher.

## Abbreviations

GABA_A_R: γ-aminobutyric acid-A receptor
NMDAR: anti-N-methyl-D-aspartate receptor
AMPAR: α-amino-3-hydroxy-5-methyl-4-isoxazole-propionic acid receptor
LGI1: leucine-rich glioma inactivated 1
Caspr2: contactin-associated protein-like 2
GABA_B_R: γ-aminobutyric acid-B receptor
DPPX: dipeptidyl-peptidase-like protein-6
GlyR: glycine receptor
mGluR5: metabotropic glutamate receptor 5
D2R: dopamine-2 receptor
AQP4: aquaporin-4
MOG: myelin oligodendrocyte glycoprotein
GFAP: glial fibrillary acidic protein
MRI: Magnetic Resonance Imaging
FLAIR: Fluid attenuated inversion recovery
EEG: electroencephalogram
MMSE: Mini-Mental State Examination
MoCA: Montreal Cognitive Assessment
MRS: Modified Rankin Scale
AZP: Azathioprine
AEDs: anti-epilepsy drugs
CTX: cyclophosphamide
GTCS: generalized tonic-clonic seizures
CSF: cerebrospinal fluid
IVIg: intravenous immunoglobulin
MTP: methylprednisolone.
