# Supplementary figures and images for: Anti-γ-aminobutyric acid-A receptor encephalitis with refractory seizures and cognitive impairment in a young woman: A case report
Source: Front Neurol. 2022 Aug 29;13:954494. doi: 10.3389/fneur.2022.954494 (PMC9465088; doi:10.3389/fneur.2022.954494)

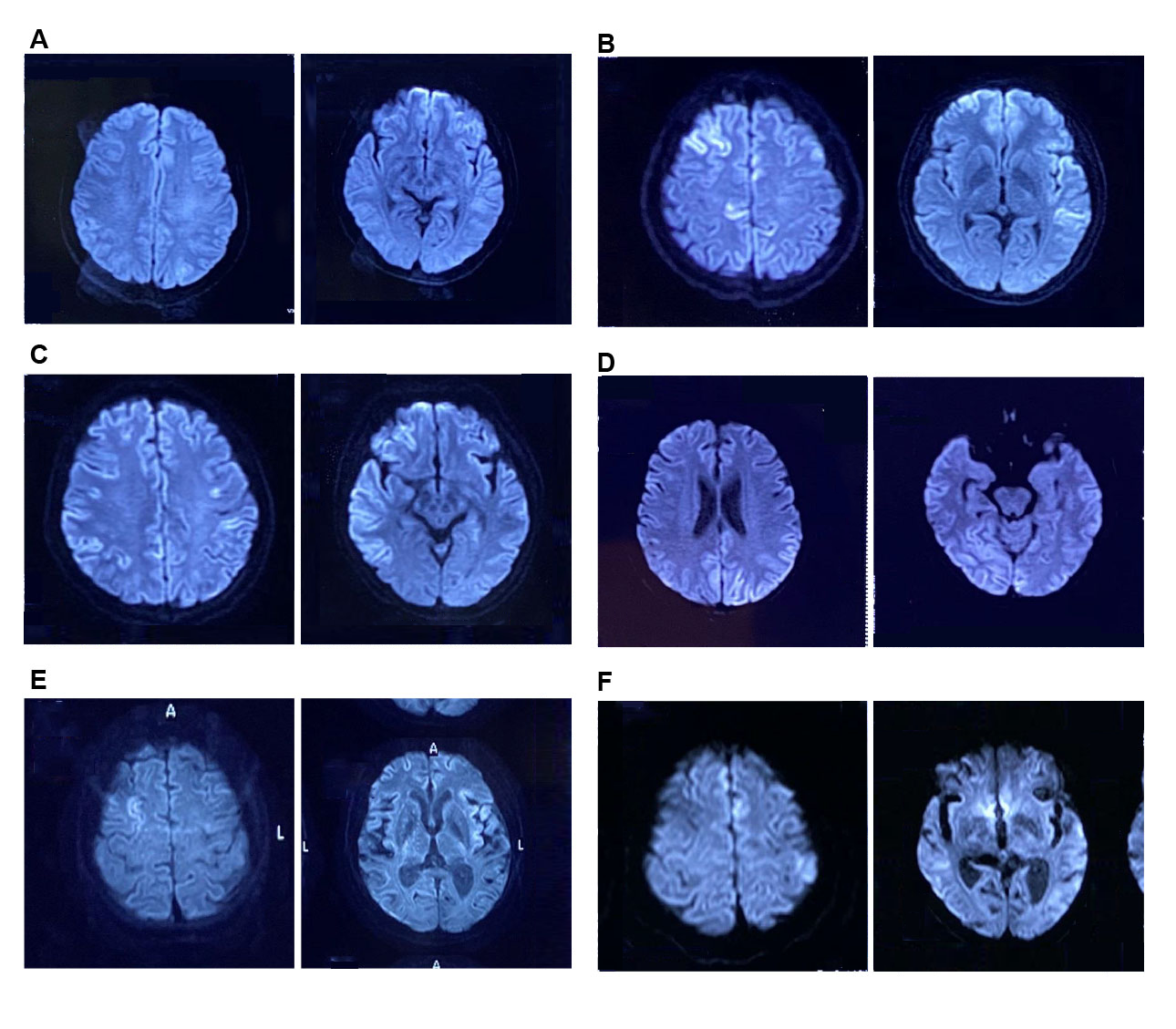

Supplement: Supplementary file 1 [file Image_1.TIF]
